# An excellent response to tofacitinib in a Brazilian adolescent patient with alopecia areata: A case report and a review of the literature

**DOI:** 10.1002/ccr3.2484

**Published:** 2019-11-21

**Authors:** Rachel Berbert Ferreira, Sineida Berbert Ferreira, Morton Aaron Scheinberg

**Affiliations:** ^1^ Faculdade de Medicina Centro Universitario Cesumar Maringá Brazil; ^2^ Private Clinic – Dermatologia Dra. Sineida Berbert Ferreira Maringa Brazil; ^3^ Hospital Israelita Albert Einstein Sao Paulo Sao Paulo Brazil

**Keywords:** adolescent, alopecia areata, dermatology, hair loss, JAK inhibitors, tofacitinib

## Abstract

Alopecia areata is a common autoimmune disease, with a negative impact in health‐related quality of life, especially when affecting children and adolescents. Current medical therapies, mainly for severe disease, are not effective. There are no FDA (Food and Drug Administration)‐ or ANVISA (Agência Nacional de Vigilância Sanitária)‐approved therapy for children with alopecia areata. JAK inhibitors are emerging as a promising therapy.

## Graphical abstract

Alopecia areata is a common autoimmune disease, with a negative impact in health‐related quality of life, especially when affecting children and adolescents. Current medical therapies, mainly for severe disease, are not effective (*Semin Cut Med Surg*, 2015; 34:72). There are no FDA (Food and Drug Administration)‐ or ANVISA (Agência Nacional de Vigilância Sanitária)‐approved therapy for children with alopecia areata. JAK inhibitors are emerging as a promising therapy.

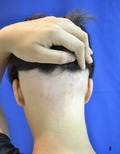

## 1 CASE REPORT

**Figure 1:**
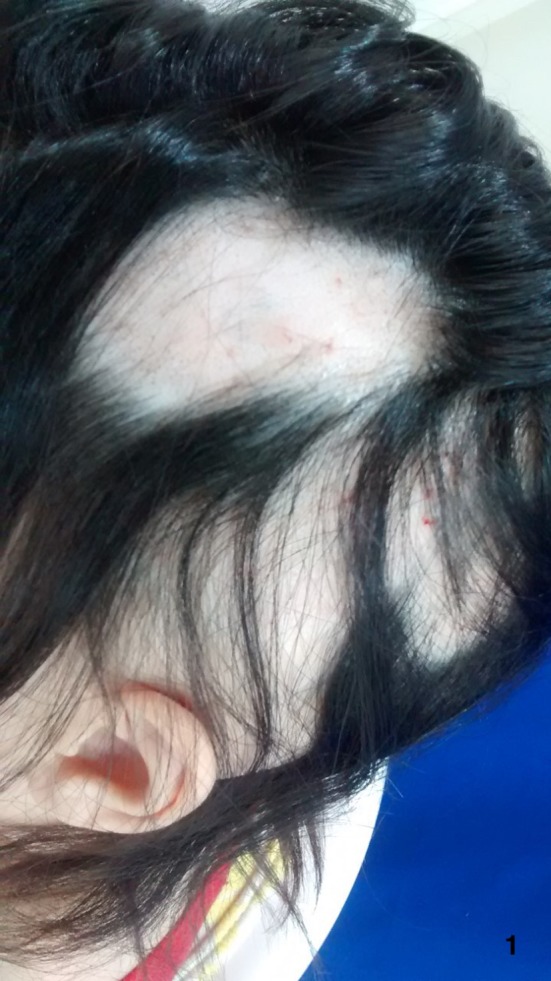
Before evolving to alopecia totalis, the patient had patches on her scalp but failed to regrowth with oral and topical treatments

**Figure 2:**
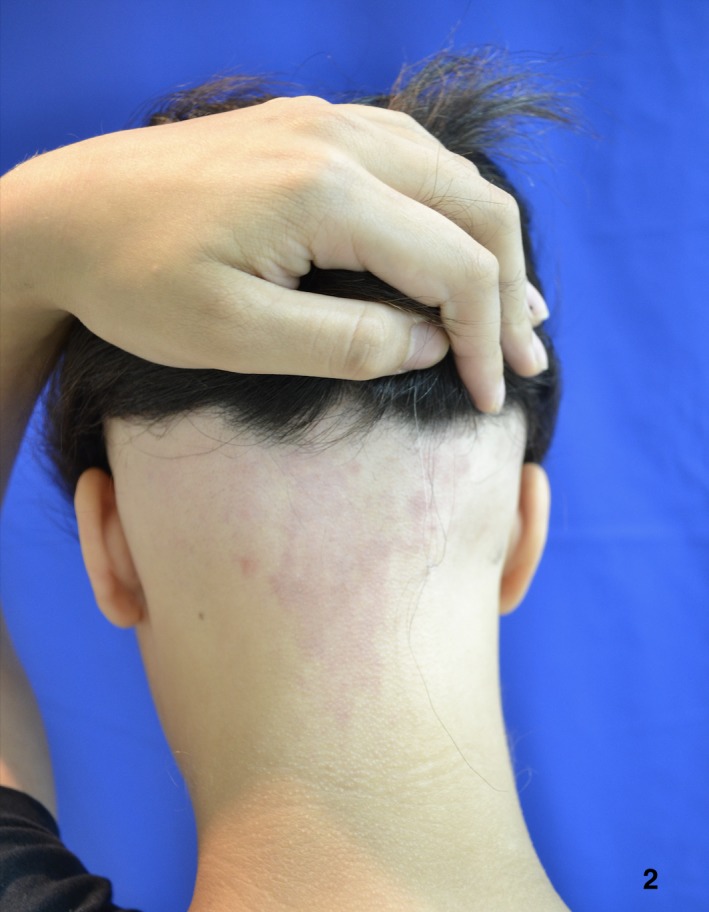
Before evolving to alopecia totalis, the patient had patches and ophiasic pattern alopecia, resistant to treatment

**Figure 3:**
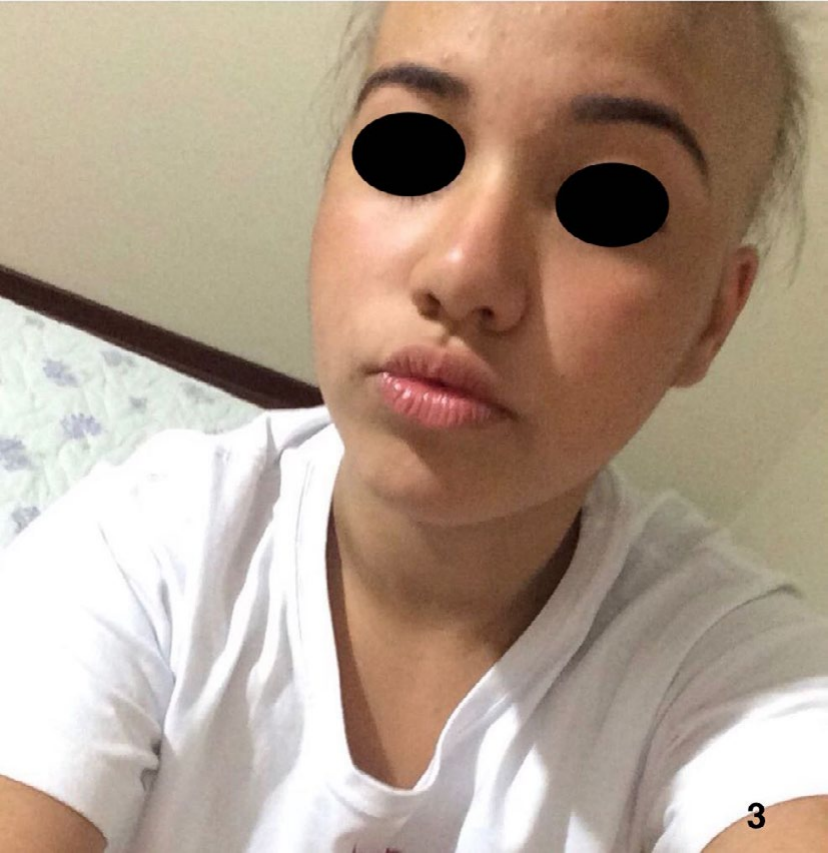
After developing alopecia totalis and right before starting treatment with tofacitinib 5 mg BID

**Figure 4:**
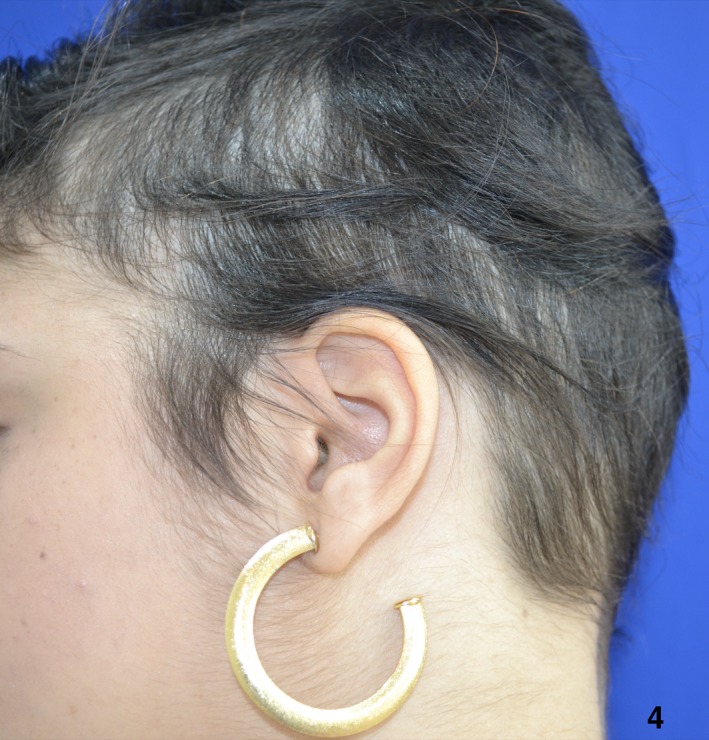
Seven months after, she started treatment with tofacitinib 5 mg BID

**Figure 5:**
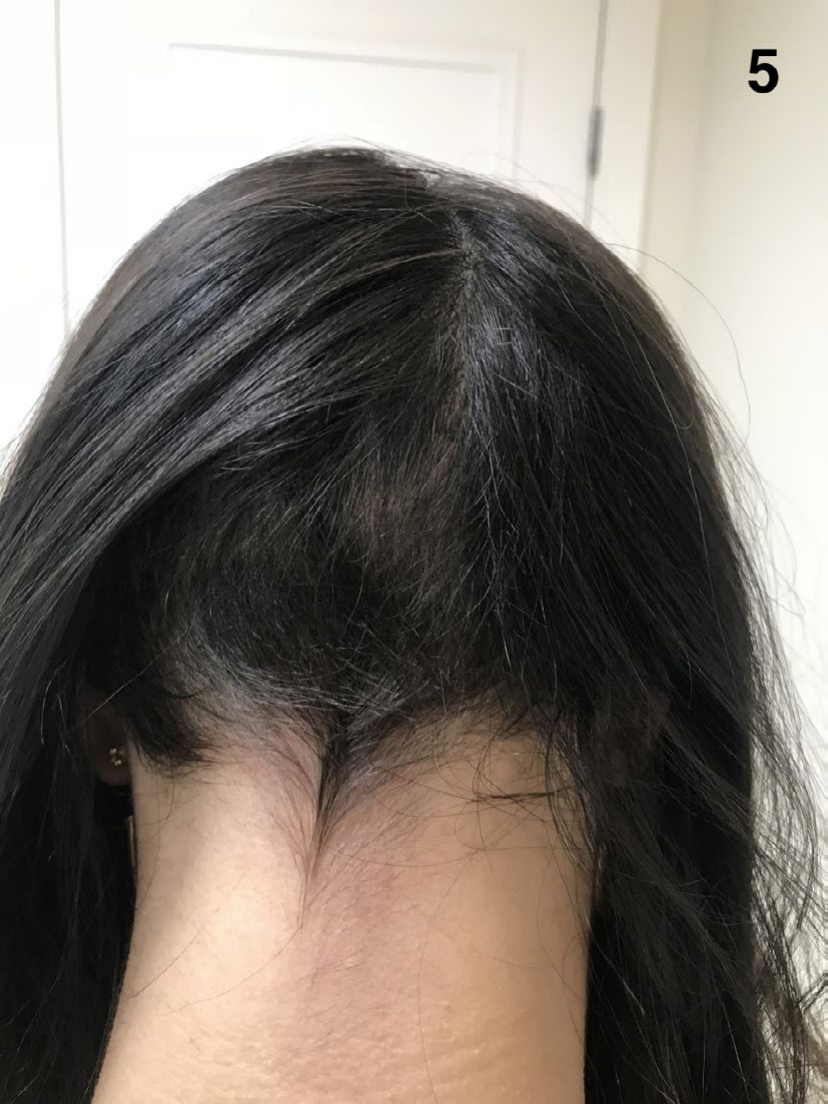
The patient had an excellent regrowth but still had an ophiasic pattern. After almost 1 y of treatment, she started to regrowth hair on the occipital region as well

**Figure 6:**
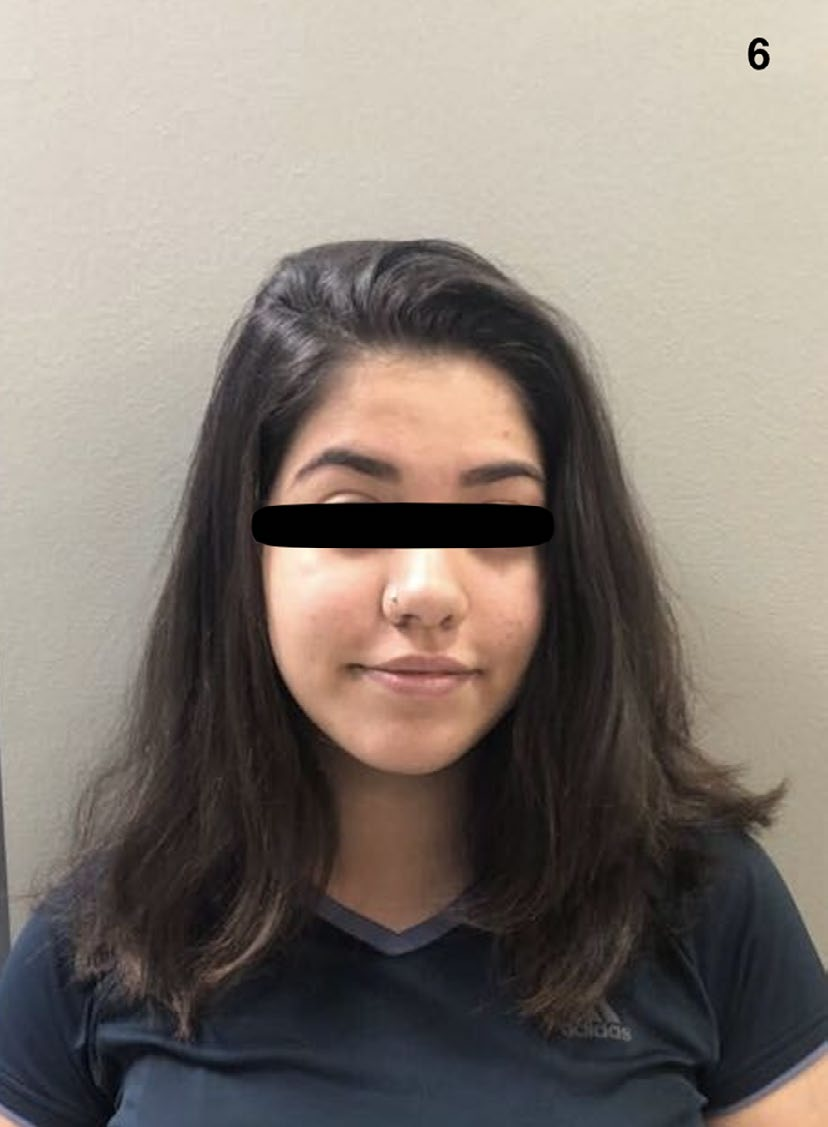
After 19 mo of treatment with tofacitinib 5 mg BID

**Figure 7:**
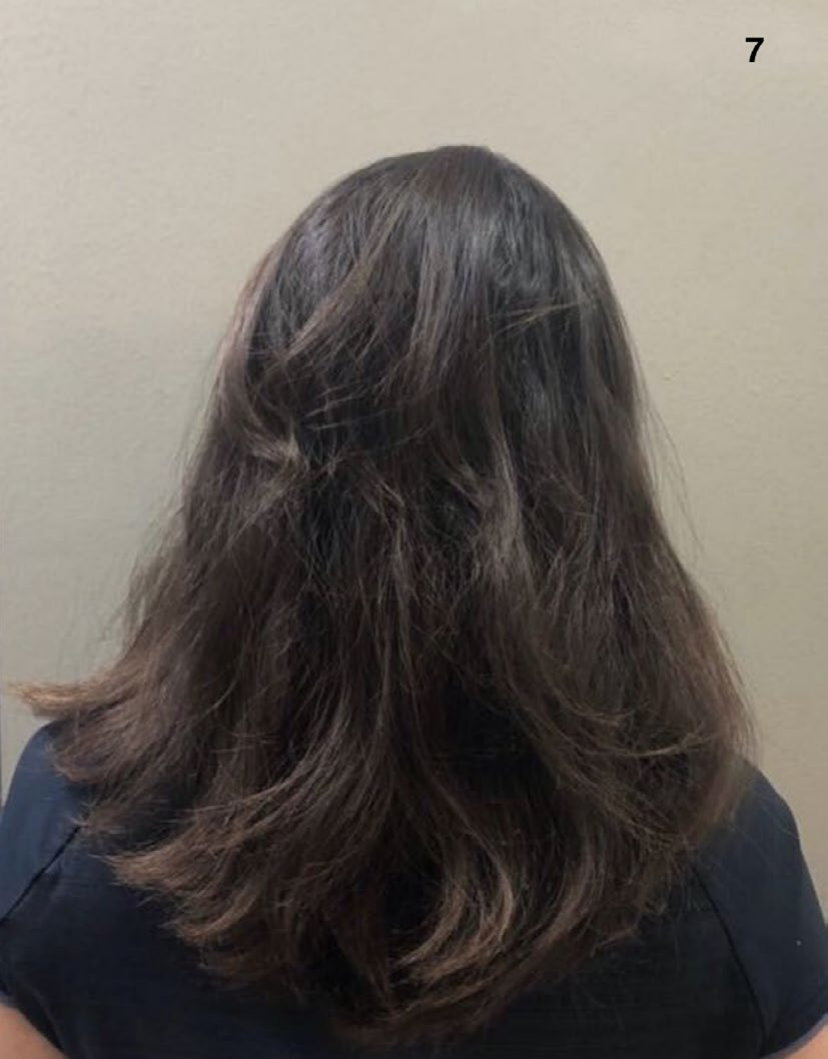
After 19 mo of treatment with tofacitinib and full regrowth, including on the occipital region

The case presented was a 13‐year‐old white woman, with 5‐year history of persistent severe alopecia areata: When she was 8 years old, she began with patches on her scalp and then developed alopecia totalis, including some patches on her arms and legs. Eyelashes, eyebrows, and nails were not affected. Previous treatments on AA refractory with their results are as follows: topical betamethasone: 0.05%— no improvement; monthly cycles of oral prednisone: 0.5‐0.8 mg/kg/d for over 5 years, produced some adverse effects like acne and weight gain; oral methotrexate 10 mg once a week followed by folic acid the next day during 9 months—no response; oral cyclosporine 3 mg/kg/d during 4 months—no improvement; and intralesional acetonide triamcinolone—good response, but resulted in severe alopecia. It was painful, and we were not able to use it all over her scalp. In some areas of the scalp, like the occipital region, there was no response to any treatment, even intralesional corticosteroids. Dermoscopy was carried out before the treatment with a Dermlite II Hybrid, with the dermoscopic features showing yellow and black dots, broken hairs, and exclamation mark hairs, representing sign of disease activity. The following baseline evaluation using laboratory tests was performed: complete hemogram, liver function test, renal parameters, lipids, Mantoux test, hepatitis B and hepatitis C, and HIV serology. All vaccines were made prior to the initiation of immunosuppressants and Janus kinase inhibitors. The patient had hypothyroidism and was being followed up by the endocrinologist, under oral levothyroxine and weekly oral supplementation of vitamin D, despite the normal range levels of 25 OH vitamin D. After 5 years of previous treatment with little or no response, we started administering tofacitinib 5 mg/BID and the patient was followed up every 4 weeks. Significant hair growth was evident at the end of 4th month. No side effects were observed. After 1 year of therapy, she had total hair regrowth. Our patient is still under treatment with tofacitinib 5 mg/BID after 19 months of therapy (Figures 1, 2, 3, 4, 5, 6, 7).

Alopecia areata is an autoimmune disease, characterized by not only scarring hair loss but dents in nails. AA is commonly associated with other diseases, such as vitiligo, rheumatoid arthritis, thyroid disease, atopic dermatitis, pernicious anemia, and diabetes.^1^ AA can affect any age range, with children representing 25% of the total cases. Triggers such as stress, infection, trauma, and hormones are known to worsen the disease in genetic predisposed individuals.

Although it is not completely elucidated, the main pathogenic event in AA is the breakdown of immune privilege in the hair follicle. The immune mechanism is mediated by cytotoxic lymphocytes and is reversed by JAK inhibition.^2^ Janus kinase protein tyrosinase family (JAK 1,2,3, and Tyk2) regulated interferon‐g in CD8 + lymphocytes. There is an accumulation of CD8+\CD4 + lymphocytes and antigen‐presenting cells around hair follicles.

The discovery that blocking the common signaling JAK pathway could reverse alopecia areata and promote remission of induced inflammatory and subsequent hair regrowth was initially observed by Jabbari et al, in 2012.

Tofacitinib citrate is a Jak 1/3 inhibitor, which was initially approved by FDA for the treatment of rheumatoid arthritis. After a first case report by Craiglow and King^3^ showing a patient with psoriasis and alopecia areata who had complete hair growth, when treated with tofacitinib, some other cases were also reported using it.^4^, ^5^, ^6^, ^7^

Then, clinical trials were performed to assess safety and efficacy of tofacitinib in patients with severe AA, AT, and AU.^8^ On the other hand, there are only few studies in children and adolescents.^9^ One review of Craiglow et al showed 13 adolescents with AA totalis or universalis, treated with tofacitinib, where 70% achieved full regrowth with no serious adverse effects.^10^ Leslie Castelo‐Soccio reported eight patients on tofacitinib, and all of them had significant hair regrowth, although no patient achieved 100 percent regrowth of scalp hair. Contrary to the series reported, our patient had complete hair growth on the scalp. The same group described experience with topical 2% tofacitinib, for patients that were not able to show complete hair growth only with oral tofacitinib, but results were mixed with some showing partial response and some with better response after subsequent use of oral formulation.^11^, ^12^ Very recently, Craiglow and King reported on four preadolescents (8‐10 years old) that were treated with oral tofacitinib, and all of them had previously multiple‐failed treatments with favorable responses. Safety in this younger group will need a longer follow‐up since no established literature is yet available. One of the issues to be established is duration of treatment and risks of relapse after cessation of therapy something that awaits guidelines derived from real‐life and clinical trial experience. The results observed in our patient and the previous three reports here described encourage us to offer this alternative for refractory adolescent patients. The case reported in this study is the youngest patient ever reported to be successfully treated with oral tofacitinib in Brazil for alopecia areata and its variants.

## CONFLICT OF INTEREST

None declared.

## AUTHORS CONTRIBUTIONS

Sineida Berbert Ferreira: involved in direct management of the patient. Rachel Berbert Ferreira: reviewed the pertinent literature, the manuscript, and the final draft. Morton Scheinberg: drafted the manuscript and supervised the final draft.
